# Long-term efficacy and safety of subcutaneous pasireotide in acromegaly: results from an open-ended, multicenter, Phase II extension study

**DOI:** 10.1007/s11102-013-0478-0

**Published:** 2013-03-26

**Authors:** Stephan Petersenn, Andrew J. Farrall, Christophe Block, Shlomo Melmed, Jochen Schopohl, Philippe Caron, Ross Cuneo, David Kleinberg, Annamaria Colao, Matthieu Ruffin, Karina Hermosillo Reséndiz, Gareth Hughes, Ke Hu, Ariel Barkan

**Affiliations:** 1 ENDOC Center for Endocrine Tumors, Altonaer Str. 59, 20357 Hamburg, Germany; 2 Brain Research Imaging Centre, Western General Hospital, University of Edinburgh, Edinburgh, UK; 3 Department of Endocrinology, Diabetology and Metabolism, Antwerp University Hospital, Antwerp, Belgium; 4 Division of Endocrinology and Metabolism, Cedars-Sinai Medical Center, Los Angeles, CA USA; 5 Medizinische Klinik und Poliklinik IV, Campus Innenstadt, University of Munich, Munich, Germany; 6 Service d’Endocrinologie, Maladies Métaboliques et Nutrition, Centre Hospitalier Universitaire Larrey, Toulouse, France; 7 Department of Diabetes and Endocrinology, Princess Alexandra Hospital, Brisbane, Australia; 8 Neuroendocrine Unit, New York University School of Medicine, New York, NY USA; 9 Dipartimento di Medicina Clinica e Chirurgia, Università Federico II di Napoli, Naples, Italy; 10 Novartis Pharma AG, Basel, Switzerland; 11 Novartis Pharmaceuticals, East Hanover, NJ USA; 12 Pituitary and Neuroendocrine Center, University of Michigan, Ann Arbor, MI USA

**Keywords:** Acromegaly, Efficacy, Pasireotide, Safety, SOM230, Somatostatin analogue

## Abstract

Pasireotide has a broader somatostatin receptor binding profile than other somatostatin analogues. A 16-week, Phase II trial showed that pasireotide may be an effective treatment for acromegaly. An extension to this trial assessed the long-term efficacy and safety of pasireotide. This study was an open-label, single-arm, open-ended extension study (primary efficacy and safety evaluated at month 6). Patients could enter the extension if they achieved biochemical control (GH ≤ 2.5 μg/L *and* normal IGF-1) or showed clinically relevant improvements during the core study. Thirty of the 60 patients who received pasireotide (200–900 μg bid) in the core study entered the extension. At extension month 6, of the 26 evaluable patients, six were biochemically controlled, of whom five had achieved control during the core study. Normal IGF-1 was achieved by 13/26 patients and GH ≤ 2.5 μg/L by 12/26 at month 6. Nine patients received pasireotide for ≥24 months in the extension; three who were biochemically controlled at month 24 had achieved control during the core study. Of 29 patients with MRI data, nine had significant (≥20 %) tumor volume reduction during the core study; an additional eight had significant reduction during the extension. The most common adverse events were transient gastrointestinal disturbances; hyperglycemia-related events occurred in 14 patients. Twenty patients had fasting plasma glucose shifted to a higher category during the extension. However, last available glucose measurements were normal for 17 patients. Pasireotide has the potential to be an effective, long-term medical treatment for acromegaly, providing sustained biochemical control and significant reductions in tumor volume.

**Electronic supplementary material:**

The online version of this article (doi:10.1007/s11102-013-0478-0) contains supplementary material, which is available to authorized users.

## Introduction

Acromegaly is a serious disorder of growth hormone (GH) hypersecretion that usually develops over many years. In more than 90 % of patients, the excessive GH level is produced by a pituitary somatotroph adenoma [1]. GH induces synthesis of insulin-like growth factor 1 (IGF-1) via hepatic GH receptors; elevated levels of GH and IGF-1 can lead to metabolic dysfunction and somatic growth [2]. Control of GH and IGF-1 is important in patients with acromegaly if mortality is to be reduced to expected levels [3]. Transsphenoidal surgery is the recommended first-line treatment for many patients with acromegaly, particularly those with microadenomas [4, 5]. However, 40–60 % of patients with acromegaly will experience persistent or recurrent disease following surgery, necessitating additional therapy [1, 4, 6].

Somatostatin analogues are the first-line medical treatment for patients with acromegaly. Compared with the universal somatostatin receptor subtype (sst) binding of somatostatin, the currently available somatostatin analogues octreotide and lanreotide act preferentially at the somatostatin receptor sst_2_ [7–9]. However, pituitary tumors in patients with acromegaly express both sst_2_ and sst_5_ [10]. A somatostatin analogue with a broader binding profile than octreotide or lanreotide may therefore provide additional therapeutic benefits.

Pasireotide (SOM230) is a multireceptor-targeted somatostatin analogue with a unique receptor binding profile, having high affinity for sst_2_ and sst_5_, as well as sst_1_ and sst_3_ [7, 10]. Therefore, it has the potential to be a more effective therapy for acromegaly than octreotide or lanreotide [11]. In a 16-week, Phase II study of 60 patients with active acromegaly, biochemical control of GH and IGF-1 levels was achieved by 27 % of patients after 12 weeks of treatment with subcutaneous (sc) pasireotide, and 39 % of patients achieved significant (≥20 %) tumor volume reduction [12]. This paper presents results from the open-ended extension period of this Phase II study and evaluates the long-term efficacy and safety of pasireotide in patients with acromegaly.

## Materials and methods

### Study design

This open-label, multicenter, single-arm extension study (ClinicalTrials.gov NCT00171730) was a planned extension to a 16-week, multicenter, randomized, open-label, crossover Phase II study (the core study; ClinicalTrials.gov NCT00088582) [12]. During the core study, patients (n = 60) received 4 weeks’ treatment with octreotide 100 μg sc tid followed by 12 weeks’ treatment with pasireotide 200, 400 and 600 μg sc bid for 1 month each in random order. To be included in this extension, patients either achieved biochemical control after at least 1 month of pasireotide at any of the three doses in the core study or achieved clinical benefit as determined by the investigator. Patients who achieved biochemical control during the core study initiated therapy in the extension study with the lowest pasireotide dose which provided biochemical control. Patients who did not achieve biochemical control but experienced clinical benefit during the core study initiated therapy in the extension study with pasireotide 600 μg sc bid. Dose adjustments were permitted in response to suboptimal biochemical control, with a maximum permitted dose of pasireotide of 900 μg sc bid.

During the extension study, pasireotide was administered for as long as treatment benefit was derived. Patients were discontinued from the extension study if they received any other therapeutic intervention for the treatment of acromegaly, failed to demonstrate continued benefit from pasireotide, experienced any unacceptable adverse event (AE) or were not compliant with the dosing schedule. Therefore, end of study was unique for each patient.

This study was approved by the Independent Ethics Committee, Institutional Review Board or Research Ethics Board (IEC/IRB/REB) for each study center. The study was conducted according to the ethical principles of the Declaration of Helsinki. All patients provided written informed consent.

### Patients

Patients aged 18–80 years who completed the core study were eligible to enter the extension phase if they had achieved biochemical control (GH ≤ 2.5 μg/L and normalized IGF-1) after at least 1 month of pasireotide administration at any of the three doses, or if they showed clinically relevant improvement according to investigator judgment from at least one of the pasireotide regimens. Patients who experienced any unacceptable AEs, who experienced tumor compression of the optic chiasm causing visual-field defects, or who required surgical intervention as a result of tumor compression during the core study were excluded from the extension study. Patients were also excluded if they developed impaired glucose tolerance or diabetes mellitus during the core study that did not resolve when pasireotide treatment was stopped (three patients were excluded from entering the extension study due to diabetes mellitus or hyperglycemia), or if they experienced cardiovascular morbidity (e.g. congestive heart failure, unstable angina), symptomatic cholelithiasis or liver disease during the core study.

### Endpoints

The primary efficacy evaluation was the percentage of patients achieving biochemical response defined as GH level ≤2.5 μg/L *and* normal IGF-1 (age and sex matched) at month 6 in the extension study. The proportions of patients with control of GH alone or IGF-1 alone were also recorded. GH and IGF-1 levels were evaluated every 3 months throughout the extension study. To calculate mean circulating GH levels, blood samples were taken at 30, 60, 90 and 120 min after the morning administration of pasireotide. IGF-1 levels were calculated as the mean of two measurements taken 30 and 1 min before pasireotide administration.

Pituitary magnetic resonance imaging (MRI) scans were performed at the screening visit before the core study (pre-octreotide), at the end of the core study (after 3 months of pasireotide), and at the last visit or every 6 months during the extension study. Digital images were assessed using established techniques and criteria by a central neuroradiologist. Significant tumor response was defined as a ≥20 % decrease in tumor volume.

Acromegaly symptom severity was recorded. The severity of headache, perspiration, paresthesia, fatigue, osteoarthralgia and carpal tunnel syndrome was recorded on a five-point scale (0 = absent, 1 = mild, 2 = moderate, 3 = severe, 4 = very severe). Sleep apnea was assessed using the Epworth Sleepiness Scale [13], which assesses the level of sleepiness in different situations on a four-point scale (0 = would never doze, 1 = slight chance of dozing, 2 = moderate chance of dozing, 3 = high chance of dozing).

Safety and tolerability were assessed every 3 months. The primary safety evaluation was based on 6 months of pasireotide treatment and reported annually thereafter. Assessments included AEs, laboratory findings, electrocardiogram (ECG) recordings and gallbladder ultrasound. AEs were classified according to severity (Common Terminology Criteria for Adverse Events, CTCAE v3.0 [14]) and relationship with study drug and were reported by preferred term.

### Hormone assays

Serum GH and IGF-1 were measured by a central laboratory using validated chemiluminescent immunometric assays (DPC Immulite 2000; Diagnostic Products Corp., Los Angeles, CA). The lower limit of detection for GH was 0.1 μg/L, with intra- and inter-assay coefficients of variation ≤10 %. For IGF-1, the lower limit of detection was 25 μg/L, with intra- and inter-assay coefficients of variation ≤8.3 %. Normal values for IGF-1 were 182–780, 114–492, 90–360 and 71–290 μg/L in patients aged 16–24, 25–39, 40–54, and ≥55 years, respectively.

### Pharmacokinetics

Blood samples for pharmacokinetic assessments were collected at each scheduled visit during GH assessment (1 min pre-dose and 1 and 2 h post-dose). Plasma concentrations of pasireotide were measured using a validated radioimmunoassay with a lower limit of detection of 0.15 ng/mL.

### Statistical methods

Efficacy analysis was based on patients who had at least two GH measurements and both pre-dose IGF-1 measurements at the corresponding visit. The safety population included all patients who had received pasireotide sc in the extension study.

Results from all centers were combined. Response rates and 95 % confidence intervals (CI) were provided by dose.

## Results

### Patients

Thirty of the 60 patients who received pasireotide in the core study entered the extension. The core and extension studies had individual study protocols, resulting in an interphase dosing holiday of variable length depending on study site. Six patients had no dosing holiday, three patients received octreotide sc treatment between the end of the core study and entering the extension, and 21 patients received no treatment between the end of the core study and entering the extension [the mean duration of the dosing holiday was 43.6 days in these patients (range 12–220 days)].

The median age of the patients enrolled in the extension study was 45.1 years (Table 1). Females comprised 53 % of the extension study population, compared with 45 % in the core study. Before enrollment in the core study, 73 % of extension patients received treatment with somatostatin analogues and 63 % underwent pituitary surgery for acromegaly (these values were 67 and 58 %, respectively, in the core study population). Thirteen percent of patients in the extension were de novo and had not received prior medical, radiation or surgical treatment for acromegaly (Table 1); the corresponding proportion for the core study was 23 %.

**Table 1:** Summary of core baseline demographics and baseline characteristics of all patients receiving at least one dose of pasireotide in the extension study

	Overall population (n = 30)
Age (years)	
Median (range)	45.1 (21–84)
Sex	
Female, n (%)	16 (53.3)
Race, n (%)	
Caucasian	26 (86.7)
Other	4 (13.3)
Previous treatment for acromegaly, n (%)	
De novo^a^	4 (13.3)
Previous somatostatin analogue treatment	22 (73.3)
Previous surgery	19 (63.3)
Previous radiotherapy	6 (20.0)
Time since diagnosis (years)^b^	
<1	5 (16.7)
1 to <5	12 (40.0)
5 to <10	7 (23.3)
≥10	6 (20.0)

^a^No prior medical, radiation or surgical treatment for acromegaly

^b^Time between diagnosis of acromegaly and the beginning of the core study

Of the 30 patients who entered the extension, eight discontinued because of an unsatisfactory therapeutic effect, four discontinued because of AEs (three for hyperglycemia and one for colon cancer and gallbladder polyp), and three withdrew consent. An additional eight patients discontinued in order to enroll in another pasireotide study (NCT00446082, CSOM230C2110). The median duration of treatment was 22.7 months (range 4–61 months).

### Effects of pasireotide on GH and IGF-1 levels

A wide dose range from 400 to 1,800 μg daily was observed across the 30 patients who entered the extension. However, for individual patients, the extent of dose titration in the extension was relatively small.

After 3 months of treatment with pasireotide sc (end of core study), the mean ± SD GH level was 8.2 ± 10.1 μg/L, with a mean change (±SD) in GH from pasireotide sc baseline of −1.1 ± 11.5 μg/L (n = 27 evaluable patients). By month 21 of the extension, the mean GH level had decreased to 4.9 ± 5.8 μg/L, a mean (±SD) change from pasireotide sc baseline of −1.7 ± 5.6 μg/L (12 evaluable patients). The mean IGF-1 level was 612 ± 209 μg/L at the pasireotide sc core study baseline and 671 ± 201 μg/L after 3 months [end of core study (n = 30 for both)]. At month 21 of the extension, the mean IGF-1 level had decreased to 296 ± 112 μg/L (n = 12), a mean (±SD) change from baseline of −279 ± 160 μg/L.

In the primary efficacy analysis at month 6 of the extension, six of the 26 evaluable patients [with at least two GH measurements and two predose IGF-1 samples (23 %; 95 % CI, 9.0–43.6 %)] had full biochemical control (defined as GH ≤ 2.5 μg/L and age- and sex-normalized IGF-1). Five of these patients had already achieved biochemical control during the core study, and one patient achieved control for the first time during the study. Of the six patients with full biochemical control, four were receiving pasireotide 1,200–<1,500 μg/day during the study, one was receiving pasireotide <1,200 μg/day, and one was receiving pasireotide ≥1,500 μg/day. At month 6 of the extension, 50 % of evaluable patients (13/26) had normal IGF-1 levels, four of whom achieved control for the first time during the extension. A similar proportion of patients (46 %, 12/26) had GH ≤ 2.5 μg/L at month 6, one of whom achieved control for the first time during the extension study.

Nine patients continued in the extension study for at least 24 months. At month 24, over half of the patients (56 %, 5/9) had normal IGF-1 and the same number had GH ≤ 2.5 μg/L. Three patients (33 %) achieved biochemical control of both GH and IGF-1 levels, all of whom were receiving a daily pasireotide dose of between 1,200 and 1,500 μg.

### Effect of pasireotide on tumor volume

Of the 30 patients who entered the extension study, 29 underwent a baseline MRI on entry into the core study. Of these 29 patients, 17 (58.6 %) had achieved a significant (≥20 %) tumor volume reduction from core baseline to last assessment. Of the 20 patients without significant tumor volume reduction before the start of the extension study, five subsequently experienced significant tumor volume reduction by month 6, and an additional two patients achieved significant tumor volume reduction by month 24.

**Fig. 1:**
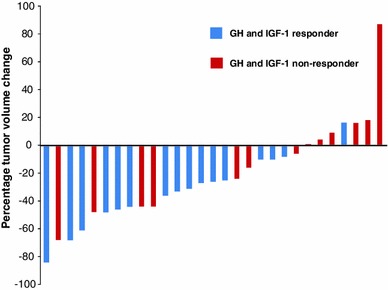
Percentage change in pituitary tumor volume from core study baseline to the last extension study visit (n = 29). The GH and IGF-1 responders shown here are those who were responders at any time point

No patient had a ≥20 % increase in tumor volume during the core study, and none of the patients enrolled in the extension study had a ≥20 % increase between core baseline and month 6 of the extension study. At month 6 of the extension study, two (50.0 %) of the four patients with full biochemical control and two (18.2 %) of the 11 patients without full biochemical control had a ≥20 % decrease in tumor volume from extension baseline. Between core baseline and the last available assessment in the extension study, 12/16 (75.0 %) responders and 5/13 (38.5 %) non-responders had at least a ≥20 % decrease in tumor volume (Fig. 1) and one non-responder had a ≥20 % increase in tumor volume. Of the five non-responders who had a ≥20 % decrease in tumor volume, three had decreases in GH and IGF-1 from core baseline.

**Fig. 2:**
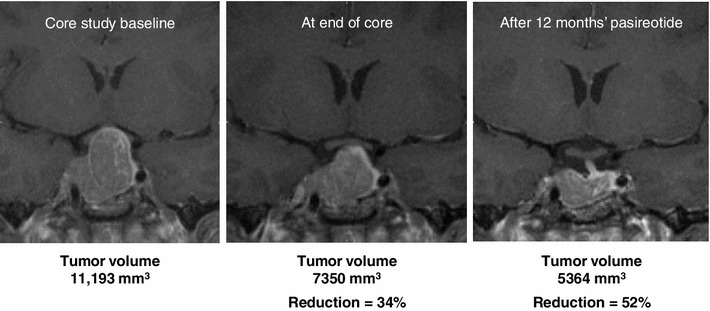
Coronal T1 MR images of a patient, illustrating significant tumor volume reduction during treatment with pasireotide

Figure 2 shows the MR images of a patient with tumor volume reduction in the core study who achieved further tumor volume reduction with extended pasireotide treatment.

### Effect of pasireotide on symptoms of acromegaly

**Fig. 3:**
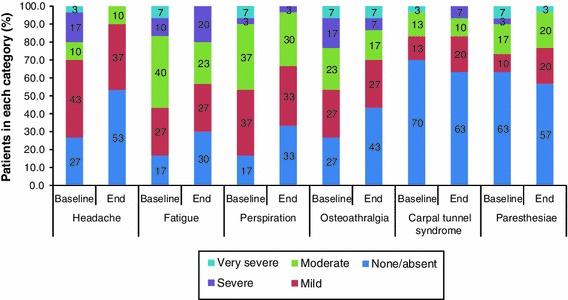
Summary of acromegaly symptom severity at extension baseline and end of the extension study

During the extension study, the number of patients with an absence of headache, fatigue, perspiration and osteoarthralgia approximately doubled (Fig. 3). For each of the symptoms assessed, more than 50 % of patients experienced no or mild symptoms at their last available assessment. No patients had very severe headache, fatigue or perspiration at their last available assessment. There were no clear changes in the severity of carpal tunnel syndrome or paresthesia over time.

### Pharmacokinetics

Plasma concentrations of pasireotide typically peaked at 1 h post-dose, based on the data points available pre-dose and 1 and 2 h post-dose. The dose-normalized trough concentration–time profiles from individual patients are shown in Supplemental Fig. 1. Pasireotide exposure tended to be stable over time in most patients.

### Safety and tolerability

All patients experienced at least one AE that was considered related to the study drug. Most AEs were mild to moderate in severity and resolved spontaneously during continued treatment with pasireotide. AEs that were suspected to be drug related were generally those expected for a somatostatin analogue in this patient population, i.e. mostly transient gastrointestinal disturbances, with the exception of the degree of hyperglycemia-related AEs (Table 2).

**Table 2:** AEs (by preferred term) with a suspected study drug relationship reported in ≥5 % of patients

	Overall patient population
	N = 30
	n (%)
Diarrhea	14 (46.7)
Nausea	10 (33.3)
Abdominal pain	6 (20.0)
Flatulence	6 (20.0)
Diabetes mellitus	5 (16.7)
Dizziness	5 (16.7)
Cholelithiasis	4 (13.3)
Type 2 diabetes mellitus	4 (13.3)
Increased blood glucose	3 (10.0)
Esophageal spasm	2 (6.7)
Fatigue	2 (6.7)
Hypoglycemia	2 (6.7)
Arthralgia	2 (6.7)
Paresthesia	2 (6.7)

In total, hyperglycemia-related AEs were reported in 12 patients (suspected of study drug relationship), nine of whom experienced more than one hyperglycemia-related event. Of the five patients who experienced AEs of diabetes mellitus, four had a previous history of glucose disorders. The majority of the hyperglycemia-related events were mild to moderate in severity and were managed with appropriate treatment.

No deaths occurred during the study. Seven patients experienced serious AEs (SAEs), two of which were considered related to study drug (worsening of diabetes mellitus and cholecystitis). One patient with pre-existing diabetes mellitus had an SAE of worsening diabetes mellitus, which was thought to be related to the study drug and which required insulin therapy. Two patients had SAEs of new-onset hyperglycemia, which was not suspected to be related to the study drug. Four patients discontinued pasireotide because of AEs: three because of hyperglycemia or diabetes mellitus, and one because of cholecystitis and colon cancer. Two of the observed cases of hyperglycemia or diabetes mellitus that led to discontinuation were thought to be related to the study drug, as was the cholecystitis.

The mean ± SD fasting plasma glucose (FPG) level was 5.36 ± 0.96 mmol/L (96.6 ± 17.3 mg/dL) at extension baseline, 5.86 ± 1.54 mmol/L (106 ± 27.7 mg/dL) at month 6, and 5.86 ± 2.45 (106 ± 44.1 mg/dL) at month 9 of the extension. Most patients (n = 20) exhibited a shift to a higher FPG category at some point during the extension study, although many returned to the same category or improved category by their last visit. Of the 17 patients who had an FPG level <5.6 mmol/L (<100 mg/dL, normal glucose tolerance) at extension baseline, 11 patients had a shift to a higher FPG category at some point during the extension; eight had their highest recorded FPG level between 5.6 and 6.9 mmol/L (100 and <126 mg/dL, impaired fasting glucose), and three had their highest level at ≥7.0 mmol/L (≥126 mg/dL, diabetes mellitus). When looking at their last available value, 13 of the 17 patients had normal glucose, three had impaired fasting glucose and one had diabetes mellitus (Table 3). Twelve patients had impaired fasting glucose at extension baseline. Of these, nine had their highest recorded value at ≥7.0 mmol/L (≥126 mg/dL, diabetes mellitus). Based on their last available value, one of these 12 patients remained in the impaired fasting glucose category, seven had an increase in FPG category to diabetes mellitus, and four patients had an improvement, being shifted from the impaired fasting glucose to the normal glucose category (Table 3).

**Table 3:** Shift tables of fasting plasma glucose following treatment with pasireotide sc

Baseline level	<5.6 mmol/L (<100 mg/dL) (%)	5.6 and 6.9 mmol/L (100–<126 mg/dL) (%)	≥7.0 mmol/L (≥126 mg/dL) (%)	Total (%)
Highest post-baseline value				
<5.6 mmol/L	6 (20.0)	8 (26.7)	3 (10.0)	17 (56.7)
5.6–6.9 mmol/L	0	3 (10.0)	9 (30.0)	12 (40.0)
≥7.0 mmol/L	0	0	1 (3.3)	1 (3.3)
Total	6 (20.0)	11 (36.7)	13 (43.0)	30 (100)
Last available post-baseline value				
<5.6 mmol/L	13 (43.3)	3 (10.0)	1 (3.3)	17 (56.7)
5.6–6.9 mmol/L	4 (13.3)	1 (3.3)	7 (23.3)	12 (40.0)
≥7.0 mmol/L	0	0	1 (3.3)	1 (3.3)
Total	17 (56.7)	4 (13.3)	9 (30.0)	30 (100)

Blood glucose levels were based on ADA criteria. The Total column shows the number of patients at baseline within a particular category, and the middle three columns show the number of patients within a given category on treatment

Modest increases in mean HbA_1c_ levels from baseline of the extension phase were observed during the study. HbA_1c_ levels increased from a mean ± SD value of 6.16 ± 0.56 % at extension baseline to 6.82 ± 1.51 % and 6.32 ± 0.68 % at months 6 and 9 of the extension, respectively.

At the last available assessment in the extension study, gallstones were detected in 11 patients, nine of whom had gallstones at extension baseline.

Electrocardiogram conduction intervals were generally within normal ranges at all visits. One patient had a newly occurring QTcF > 450 ms during the extension study, which decreased to ≤450 ms at subsequent ECG evaluations. Overall, 17 patients had ECGs with at least one abnormality, but in six cases the overall ECG interpretation was considered to be normal. Nine patients had at least one episode of sinus bradycardia, of whom three had sinus bradycardia at the last study visit.

## Discussion

This Phase II extension study showed that, following 9 months of pasireotide therapy (3 months in the core study and 6 months in the extension study), 23 % (6/26) of patients with acromegaly achieved biochemical control (defined as GH ≤ 2.5 μg/L and IGF-1 within age- and sex-matched limits).The response rate observed in the extension is similar to that seen in the core study (27 %) after 3 months’ pasireotide treatment [12] and is consistent with the results of earlier studies of somatostatin analogues that used this definition of biochemical control [15–17]. These earlier studies reported response rates of 17–27 % after 1 year of therapy. In the present study, 50 % of patients achieved IGF-1 normalization alone and 46 % achieved GH control alone following 9 months of pasireotide therapy. Compared with the core study, an additional five patients achieved GH and/or IGF-1 control for the first time during the extension study, which suggests that extending pasireotide treatment beyond 3 months may be required to achieve full biochemical control in certain patients.

Current treatment guidelines by the Acromegaly Consensus Group define optimal disease control as normal IGF-1 for age and sex and a GH level <1.0 μg/L from a random GH measurement [18, 19]. This current GH cut-off was defined considering modern GH assays that use the World Health Organization calibrator [18, 19]. Earlier studies measuring GH by older immunoassays indicated that a random GH level <2.5 μg/L and normal IGF-1 for age and sex is associated with normal life expectancy [3, 20]. This older GH cut-off has continued to be used in recent studies evaluating the effect of treatment in patients with acromegaly and was used in the current study as availability of modern assays with the current WHO calibrator was limited at the time of study initiation.

Consistent with previous reports on long-term somatostatin analogue therapy in acromegaly [17, 21–23], pasireotide resulted in significant and ongoing reductions in tumor volume throughout the extension phase of the study. Fifty-nine percent of patients achieved a significant (≥20 %) reduction in tumor volume over the course of the core and extension studies. Moreover, a subset of patients who experienced significant tumor volume reduction during the core study went on to achieve further reductions in tumor volume during the extension phase. This suggests that patients entering the extension period benefited from continued therapy and that long-term pasireotide treatment is effective in reducing the volume of pituitary adenomas. A significant reduction in tumor volume was more commonly observed in patients with biochemical control than in uncontrolled patients. However, a reduction of at least 20 % in tumor volume was seen in 5/13 (38.5 %) patients who did not achieve biochemical control. Additional studies that further investigate the relationship between biochemical control and tumor volume under pasireotide treatment are thus warranted.

In addition to biochemical control and reductions in tumor volume, this study showed that long-term pasireotide treatment is associated with long-term improvements in the symptoms of acromegaly, including headache, fatigue, perspiration and osteoarthralgia. This is consistent with the results of previous studies that showed significant improvements in the symptoms of acromegaly following 12 months’ treatment with octreotide LAR [24]. In association with improved detection methods and visualization of the tumor, these benefits of treatment have contributed to the improvements seen in acromegaly [25].

Adverse events were mostly similar to those encountered with conventional somatostatin analogues: predominantly mild to moderate gastrointestinal disorders that resolved during continued pasireotide treatment. Hyperglycemia-related AEs were reported in 12/30 (40 %) patients. Disorders of glucose metabolism have previously been reported during treatment of acromegaly with somatostatin analogues [26, 27], including pasireotide [12]. In addition, patients with acromegaly are known to be predisposed to changes in glucose metabolism [28], which in turn leads to a higher prevalence of diabetes mellitus and glucose intolerance compared with the general population [29].

In the present study, most patients exhibited a shift to a higher FPG category at some point during the extension study, with eight patients worsening to impaired glucose tolerance (5.6–6.9 mmol/L) and 12 patients worsening to glucose levels ≥7.0 mmol/L. However, many returned to the same category or improved by their last visit, with 57 % of patients having glucose levels <5.6 mmol/L as their last available value, the same proportion as at extension baseline. Whether these improvements in FPG category at the end of the study are due to some tachyphylaxis or due to changes in antidiabetic medication cannot be distinguished. Modest increases in HbA_1c_ levels were also observed (6.16 % at extension baseline to 6.82 % at month 6). Additional studies have been conducted to determine the underlying mechanisms of pasireotide-associated hyperglycemia and to evaluate the effects of anti-diabetic drugs on glucose metabolism in pasireotide-treated healthy volunteers [30]. These have shown that pasireotide-associated hyperglycemia is related to decreases in insulin secretion and incretin hormone response. Pasireotide appears to have minimal impact on glucagon secretion and no effect on insulin sensitivity.

In conclusion, pasireotide shows promise as a long-term medical treatment for acromegaly, providing sustained biochemical control and significant reductions in pituitary tumor volume. A randomized, multicenter Phase III trial is evaluating the efficacy of pasireotide LAR compared with that of octreotide LAR, which is the current standard of care in patients with acromegaly.

## Electronic supplementary material

Below is the link to the electronic supplementary material.

Supplementary material 1 (DOCX 56 kb)
