# Supplementary material for: Long-term efficacy and safety of subcutaneous pasireotide in acromegaly: results from an open-ended, multicenter, Phase II extension study
Source: Pituitary. 2013 Mar 26;17(2):132–40. doi: 10.1007/s11102-013-0478-0 (PMC3942632; doi:10.1007/s11102-013-0478-0)
Supplement: Supplementary file 1 — Supplementary material 1 (DOCX 56 kb) [file 11102_2013_478_MOESM1_ESM.docx]

**Supplemental Figure 1. Dose-normalized pasireotide plasma concentration–time plots at pre-dose (0 h) for individual patients (n=30).** Plots include all observed plasma concentrations at 0 min (pre-dose) from all visits in each patient regardless of the dose being administered. Before Day 112 is the core phase and after Day 112 is the extension phase
